# Simplifying ART cohort monitoring: Can pharmacy stocks provide accurate estimates of patients retained on antiretroviral therapy in Malawi?

**DOI:** 10.1186/1472-6963-12-210

**Published:** 2012-07-20

**Authors:** Hannock Tweya, Caryl Feldacker, Anne Ben-Smith, Anthony D Harries, Ryuichi Komatsu, Andreas Jahn, Sam Phiri, Jean-Michel Tassie

**Affiliations:** 1 International Union against Tuberculosis and Lung Disease, Paris, France; 2 The Lighthouse Trust, P.O. Box 106, Lilongwe, Malawi; 3 International Training and Education Center for Health (I-TECH), University of Washington, 901 Boren Avenue, Suite 1100, Seattle, Wa, 98104-3508, USA; 4 Maame Akua, 43/2/391, Lilongwe, Malawi; 5 Department of Infectious and Tropical Diseases, London School of Hygiene and Tropical Medicine, Keppel Street, London, UK; 6 Global Fund to Fight Against AIDS, Tuberculosis and Malaria, Geneva, Switzerland; 7 Department of HIV/AIDS, Ministry of Health, PO Box 30377, Lilongwe, Malawi; 8 HIV/AIDS Department, WHO, Geneva, Switzerland

**Keywords:** Antiretroviral therapy, Pharmacy stock records, Patient retention, Quality improvement

## Abstract

**Background:**

Routine monitoring of patients on antiretroviral therapy (ART) is crucial for measuring program success and accurate drug forecasting. However, compiling data from patient registers to measure retention in ART is labour-intensive. To address this challenge, we conducted a pilot study in Malawi to assess whether patient ART retention could be determined using pharmacy records as compared to estimates of retention based on standardized paper- or electronic based cohort reports.

**Methods:**

Twelve ART facilities were included in the study: six used paper-based registers and six used electronic data systems. One ART facility implemented an electronic data system in quarter three and was included as a paper-based system facility in quarter two only. Routine patient retention cohort reports, paper or electronic, were collected from facilities for both quarter two [April–June] and quarter three [July–September], 2010. Pharmacy stock data were also collected from the 12 ART facilities over the same period. Numbers of ART continuation bottles recorded on pharmacy stock cards at the beginning and end of each quarter were documented. These pharmacy data were used to calculate the total bottles dispensed to patients in each quarter with intent to estimate the number of patients retained on ART. Information for time required to determine ART retention was gathered through interviews with clinicians tasked with compiling the data.

**Results:**

Among ART clinics with paper-based systems, three of six facilities in quarter two and four of five facilities in quarter three had similar numbers of patients retained on ART comparing cohort reports to pharmacy stock records. In ART clinics with electronic systems, five of six facilities in quarter two and five of seven facilities in quarter three had similar numbers of patients retained on ART when comparing retention numbers from electronically generated cohort reports to pharmacy stock records. Among paper-based facilities, an average of 13 4 hours was needed to calculate patient retention for cohort reporting using patient registers as compared to 2.25 hours using pharmacy stock cards.

**Conclusion:**

The numbers of patients retained on ART as estimated using pharmacy stock records were largely similar to estimates based on either paper registers or electronic data system. Furthermore, less time and staff effort was needed to estimate ART patient retention using pharmacy stock records versus paper-based registers. Reinforcing ARV stock management may improve the precision of estimates.

## Background

Globally, the number of HIV-infected individuals on antiretroviral therapy (ART) grew from 400,000 in 2004 to over 5.2 million by the end of 2009 [1]. In sub-Saharan Africa alone, approximately 3.9 million HIV-infected individuals received ART by December 2009, up from about 25,000 patients in 2002 [1]. The dramatic pace of ART scale-up brings huge challenges in effective and accurate monitoring of expanding ART programs including ARV drug management. Routine, accurate monitoring of patients who start ART and, most critically, those who are retained on ART is crucial both for measuring programmatic success and monitoring drug supplies. In resource-poor settings in sub-Saharan Africa, innovative, precise, and low-cost methods are needed to effectively track patients and drug supplies.

Like many of its neighbours, Malawi’s ART program expanded rapidly. As of December 31^st^, 2009, Malawi reported a total of 271,105 patients on ART. Of 377 ART facilities, 46 registered more than 2,000 patients each and 11 sites registered more than 5,000 patients [2]. Currently, ART facilities in Malawi use simple, standardized monitoring systems to record the number of new ART initiations, continuing ART patients, and transfer-in patients every quarter. This quarterly summation is referred to by the Malawi Ministry of Health (MoH) as the *Quarterly ART Cohort Report *[3]. Despite the relative simplicity of the ART cohort reporting tools, routine accurate reporting of the numbers of patients retained on ART is complicated. To report accurately, ART facilities must know both the number of patients who ever started on ART [determined from the ART treatment cards and registers] and the outcome of all patients at the end of every quarter. In Malawi, five standardised outcomes are reported: 1) alive and on therapy (implies ART retention); 2) dead; 3) stopped treatment; 4) transferred to another facility, and 5) lost to follow-up (defined as missing a clinic appointment by two months or more). The number “alive and on therapy” in a facility is calculated as the number ever started on ART **minus** deaths, stops, transfers to other clinics, and lost to follow-up.

As the number of people initiated on life-long ART grows in Malawi, other ways of estimating ART retention are needed. Compiling the quarterly ART cohort report is very labour intensive for clinics that use paper-based registers: it requires reviewing all individual treatment cards, updating the clinic register, and aggregating patient outcomes. The time and effort needed to complete this exercise raise significant concerns about the time allocation for health workers and the quality of data collection, especially as the number patients retained on ART increases. One possibility for an improved monitoring system, piloted in Malawi and elsewhere, is an electronic patient management systems [4]. However, electronic systems require significant upfront hardware costs and well-trained personnel to monitor system performance creating barriers to nationwide scale-up. An alternate possibility for low resource settings is to use pharmacy stock records to streamline the monitoring process [5,6]. If well kept, pharmacy stock information on drug dispensation may provide an accurate measure of ART retention over a defined time period. We, therefore, piloted a study in Malawi to determine whether estimates of patient retention on ART from pharmacy stock records would reflect a similar level of precision as compared to the current estimates using either paper- or electronic-based registers. We also compared the personnel time taken to compile ART patient retention data for the cohort report using paper-based patient registers versus pharmacy stock records.

## Methods

### Sample characteristics

We used a purposeful sampling technique to select 12 ART facilities representing all levels of health delivery facilities (central hospitals, district hospitals and health centres) and both paper-based and electronic systems of data management. Six selected ART sites used paper-based systems (2 district hospitals, 1 mission hospital and 3 rural health centres) and six used a real-time electronic patient management data system (2 central hospitals and 4 district hospitals). Both paper and electronic data systems collect similar information and report the same standardized information for the ART cohort report.

The Malawi ART program uses three predominant ARV regimens: first-line - Stavudine Lamivudine Nevirapine (d4T 3TC NVP); alternative first line -Zidovudine Lamivudine Nevirapine (AZT 3TC NVP) or Stavudine Lamivudine + Efavirenz (d4T 3TC + EFV) or Zidovudine Lamivudine + Efavirenz (AZT 3TC + EFV); and second line –Zidovudine Lamivudine + Tenofovir + Lopinavir Ritonavir) (AZT 3TC + TDF + LPV/r) for adults; Didanosine + Abacavir + Lopinavir (ddl ABC LPV/r) for children. D4T 3TC NVP and AZT 3TC NVP are fixed dose combinations for first-line and alternative first line respectively.

### Data collection

We collected data from routine cohort reports of patients retained in therapy and pharmacy stock records for quarter 2, April – June 2010 and quarter 3, July – September, 2010 from ART facilities using either paper- and electronic-based systems. Facilities with electronic-based systems were mainly included in the pilot to validate ART retention estimates. We used variables from the cohort report. First, we used, *the number of new patients registered* which includes both *new ART initiations* and *transfer-in patients.* Second, we used *the number of patients retained on therapy at the end of a quarter,* calculated by recording the number of patients ever started minus those who died, stopped, transferred-out and lost to follow-up at end of the quarter. Also, the cohort report had the number of patients retained in therapy stratified by type of regimen (first- and second-line). In each paper-based site, we determined time needed to compile a cohort report from ART registers through in-person interviews with those responsible for data reporting. In sites with electronic data systems, production of the cohort report data is quick, requiring only simple, pre-set commands.

Pharmacy ARV stock data were also collected from all ART facilities using the same methods, regardless of whether paper- or electronic-based systems were used for patient management. The number of ART continuation bottles [containers of tablets for 30 days for all regimen types] were documented at the start and end of a specific quarter using pharmacy stock cards. Data for re-distribution or expiration were also recorded from pharmacy stocks and delivery receipts. These data were used to calculate the total number of bottles dispensed by the clinic to patients in the specific quarter. Although dispensing intervals varied as patients received 0.5, 1, 2, 3 or more bottles for intervals of 2 weeks, 1, 2, 3 or more months, the average rate of dispensing per patient during the quarter was assumed stable over time. The number of patients retained on therapy at the end of the quarter was calculated by taking a) the number of new patients starting ART (both ARV naïve and transfer-in patients); b) multiplying this number by 1.5 as new patients use, on average, 1.5 ARV bottles per quarter; then, c) subtracting this number from the number of bottles of ART used in three months; and finally, d) dividing this number by 3 to calculate the number of patients on ART.

### Calculating patients retained on ART using ARV stock data

### Step 1*

Number of continuation ART bottles (First line, alternative first line second line) at start of the quarter (A). Number of continuation ART bottles (First line, alternative fist line, second line) at end of the quarter (B). Number of continuation ART bottles used: A – B = (C). C is the number of bottles for follow up patients from previous quarters and those registered in the current quarter at the ART facility * this calculation includes removal and addition of stocks and continuation bottles lost because of expiry, loans, theft, etc.

### Step 2

Number of new patients starting ART in the quarter. (D) Number of continuation ART bottles used by new patients in quarter = D x 1.5 = E. E is the average number of bottles during follow-up for patients registered in the current quarter.

### Step 3

Number of continuation ART bottles used = C Number of continuation ART bottles used by new patients = E. Number of continuation bottles of ART used by follow-up patients = C – E = F. F is the number of bottles for follow-up patients from previous quarters only. Number of patients on follow-up = F divided by 3 = G.

### Step 4

Number of new patients retained on ART = D. Number of follow-up patients retained on ART = G. The total number retained on ART = D + G = H*. This final number could also be stratified according to type of regimen depending on available information.

### Data analysis

Analysis was stratified by type of data system. First, among those ART facilities with paper-based ART patient registries, the number of patients retained on ART was compared between routine cohort reports from paper-based systems and pharmacy-based records. We chose an acceptable tolerance of 5.0% above or below the routine cohort report estimates when comparing estimates as an incorrect estimate of patient numbers by more than 5.0% may result in huge over quantification and supply of ARV drugs to ART facilities. In ART facilities with electronic ART data systems, we compared patient retention as determined by electronic cohort report versus paper-based pharmacy records. In case of discrepancy of more than 5.0% between the 2 sources, we were not able to determine which system was more accurate.

In paper-based facilities, brief qualitative interviews with clinical officers and data clerks were conducted to determine time taken to compile the cohort reports.

### Ethics Approval

The study was approved by the Malawi National Health Science Research and the Ethics Advisory Group of The International Union Against Tuberculosis and Lung Disease, Paris, France.

## Results

### ART facilities

Data were collected from 12 ART facilities between April and September, 2010. The numbers of ART patients ever registered at these sites by September 2010 ranged from 1,194 at Chipini Health Centre to 13,961 at Queen Elizabeth Central Hospital. The numbers of patients retained on ART varied from 781 at Chipini Health Centre to 7,774 at Queen Elizabeth Central Hospital.

### ART retention estimates in facilities using paper-based monitoring systems

Of six ART facilities using paper-based data system, overall estimates of patients retained on ART from the cohort report versus pharmacy stock cards were similar in two of three health centres and one of three district-level hospitals in quarter two (Table 1). In quarter three, estimates from both methods showed similar numbers of patients retained across two health centres and in two of three district-level hospitals. One district ART facility started using an electronic data system in quarter three and was, therefore, excluded from quarter three analysis.

**Table 1:** Comparison of total numbers of patients retained on ART between clinic cohort reports and pharmacy stock cards data in paper-based ART facilities

	**Quarter two (April to June 2010)**			**Quarter three (July to September 2010)**		
**ART facility**	**Clinic**	**Pharmacy stock cards**	Precision (%)*	**Clinic**	**Pharmacy stock cards**	Precision (%)*
	**cohort report**			**cohort-report**		
***Health Centres***						
Bangwe	1,567	1,575	*101%*	1,689	1,676	*99%*
Chipini	779	790	*101%*	838	913	*109%*
Ndirande	1,886	1,606	*85%*	2,008	2,079	*104%*
***Total***	***4,232***	***3,971***	***94%***	***4,535***	***4,668***	***103%***
***District Hospital***						
Mchinji	2,027	2,455	*121%*	2,129	2,203	*103%*
Mangochi^**₣**^	4,519	4,962	*110%*	*-*	*-*	*-*
Malamulo	2,140	2,186	*102%*	2,216	2,188	*99%*
***Total***	***8,686***	***9,603***	***111%***	***101%***	***4,345***	***4,391***

^₣^Mangochi was excluded in quarter three analysis because of the introduction of an electronic data system. *Precision is a measure of the degree of agreement for ART retention estimates from pharmacy stock records versus clinic cohort report estimates.

Table 2 shows the level of agreement of the numbers of patients retained on first-line ARV regimens according to paper-based cohort reports and pharmacy stock records. One of three health centres had similar retention estimates in both quarters. However, none of the district ART facilities had similar number of patients on the first-line regimen in quarter two and, in quarter three, two district-level hospitals had similar ART retention estimates. Further analysis for the second-line ARV regimen was not feasible as the numbers were small and prevented meaningful comparison.

**Table 2:** Comparison of numbers of patients retained on first-line ARV regimen between clinic cohort reports and pharmacy stock cards in paper-based ART facilities

	**Quarter two (April to June 2010)**			**Quarter three (July to September 2010)**		
	**First -line regimen**			**First-line regimen**		
**ART facility**	**Clinic**	**Pharmacy stocks cards**	Precision (%)*	**Clinic**	**Pharmacy stocks cards**	Precision (%)*
	**cohort report**			**cohort report**		
***Health Centre***						
Bangwe	1,554	1,639	105%	1,675	1,676	100%
Chipini	655	788	120%	692	909	131%
Ndirande	1,841	1,605	87%	1,952	2,071	106%
***Total***	***4,050***	***4,032***	100%	*4,319*	***4,656***	108%
***District Hospital***						
Mchinji	1,995	2,222	111%	2,061	2,203	103%
Mangochi^**₣**^	4,516	4,962	110%		-	-
Malamulo	2,038	1,852	91%	2,113	2,189	99%
***Total***	***8,683***	***9,036***	***104%***	***4,345***	***4,392***	***101%***

^₣^Mangochi was excluded in quarter three analysis because of the introduction of an electronic data system. *Precisions is a measure of the degree of agreement for ART retention estimates from pharmacy stock records versus clinic cohort report estimates.

### ART retention estimates in facilities using electronic monitoring systems

Among six ART facilities with electronic data systems in quarter 2, five of the six facilities had similar estimates of patients retained on ART according to the cohort report versus pharmacy stock cards (Table 3). In quarter three, five of the seven ART facilities had similar numbers of patients retained on therapy using the cohort report versus pharmacy stock cards. Mangochi District Hospital was included in quarter three comparisons because it adopted the electronic data system.

**Table 3:** Comparison of numbers of patients retained on ART between electronically generated clinic cohort reports and paper-based pharmacy stock cards in electronic-based ART facilities

	**Quarter two (April to June 2010)**			**Quarter three (July to September 2010)**		
**ART facility**	**Clinic**^**¥**^	**Pharmacy stocks cards**	Precision (%)*	**Clinic**	**Pharmacy stocks cards**	Precision (%)*
	**Cohort report**			**cohort report**		
***District Hospital***						
Dedza	1,921	1,852	*96%*	2,001	1,955	*98%*
Martin Preuss	6,524	6,493	*100%*	7,045	6,843	*97%*
Salima	2,520	2,395	*95%*	2,675	2,429	*91%*
St Gabriel	1,656	1,548	*93%*	1,731	1,679	*97%*
Mangochi^**₣**^	-	-	*-*	4215	2,326	*55%*
***Total***	***12,621***	***12,288***	***97%***	***17,667***	***15,232***	***86%***
***Central Hospital***						
Lighthouse	6,374	6,281	*99%*	6,488	6,434	*99%*
Queens Elizabeth	7,408	7,082	*96%*	7,774	7,774	*100%*
***Total***	***13,782***	***13,363***	***97%***	***14,245***	***14,208***	***100%***

^₣^Mangochi was included in quarter three analysis because of the introduction of an electronic data system. ^¥^ Electronically generated cohort report. *Precisions is a measure of the degree of agreement for ART retention estimates from pharmacy stock records versus clinic cohort report estimates.

Table 4 shows the comparison of number of patients retained on the first-line regimen based on estimates between clinic cohort report and pharmacy stock records for quarter two and three. Three of four district-level hospitals and one central hospital had similar numbers of patients on first-line regimen in quarter two; two of five district ART hospitals and both central hospitals had similar numbers of patients on the first-line regimen in quarter three.

**Table 4:** Comparison of numbers of patients retained on first-line ARV regimen between electronically generated clinic cohort reports and paper-based pharmacy stock cards in electronic-based ART facilities

	**Quarter two (April to June 2010)**			**Quarter three (July to September 2010)**		
	**First -line regimen**			**First-line regimen**		
**ART facility**	**Clinic**^¥^	**Pharmacy stocks cards**	Precision (%)*	**Clinic**	**Pharmacy stocks cards**	Precision (%)*
	**cohort report**			**cohort report**		
**District Hospital**						
Dedza	1,868	1,852	99%	1,949	1,955	100%
Martin Preuss	6,514	6,471	99%	7,024	6,806	97%
Salima	2,436	2,395	98%	2,586	2,429	94%
St Gabriel	1,412	1,542	109%	1,447	1,679	116%
Mangochi^**₣**^	-	-	-	4,145	2,326	56%
***Total***	***12,230***	***12,260***		***17,645***	***15,195***	***86%***
**Central Hospital**						
Lighthouse	6,161	6,023	98%	6,264	6,152	98%
Elizabeth	7,253	6,840	94%	7,592	7,604	100%
***Total***	***13,414***	***12,863***	***96%***	***13,856***	***13,756***	***99%***

^₣^Mangochi was included in quarter three analysis because of the introduction of an electronic data system. ^¥^ Electronically generated cohort report. *Precisions is a measure of the degree of agreement for ART retention estimates from pharmacy stock records versus clinic cohort report estimates.

### Workload and time required to compile a cohort report

Table 5 shows time required to compile cohort reports in ART facilities that used a paper-based system. On average, facilities with more than 1,200 patients on ART took 13.4 hours to compile the report; an additional 5 hours were required for report verification by the ART supervision team. In comparison, estimation of cohort data from pharmacy stock cards took 2.25 hours on average in these same facilities. The cost associated with personnel time invested in generating the cohort reports was not included as no additional costs are incurred.

**Table 5:** Comparison of time required to compile cohort reports between paper-based cohort report andpharmacy stock records in paper-based ART facilities

		**Clinic cohort report**		**Pharmacy stocks cards**	
ART facility*	**ART patients registered**	**Staff (N)**	**Hours**	**Staff (N)**	**Hours**
Chipini	1,265	3	10	1	0.75
Bangwe	2,548	5	5	1	1.33
Ndirande	3,411	4	24	1	2.5
Malamulo	3,072	2	18	1	1
Mchinji	3,891	8	10	1	2
***Average***	***2,837***		***13.4***		***2.25***

*There were 5 ART facilities that used paper-based reporting systems in the period between July and September 2010.

## Discussion

This is one of first studies to explore the feasibility of using pharmacy ARV stock cards for estimating the number of patients retained on ART in resource-constrained settings. Four of the five ART facilities with paper-based data systems in quarter 3 had acceptable estimates of patients retained on ART as, calculated from patient registers typically used for compiling the cohort report versus estimates using pharmacy stocks records. Among those with electronic systems in both quarters two and three, five ART facilities had acceptable levels of agreement between the two estimation methods. The study also suggests that estimation of patients retained on ART using pharmacy stock data may be cost-effectiveness in terms of staff time required to compile the data.

Although most paper-based ART facilities showed similar estimates of patients retained using patient registers or pharmacy stock cards, inaccuracies of these estimates may be caused by several factors. First, the paper-based system is prone to data incompleteness. A previous study looking at the extent of data inaccuracies reported up to 24% of ART registrations missing in the paper ART registers in comparison to electronic data records [7]. Second, the variations in pharmacy-based retention estimates might be due to differences in pharmacy operations across the facilities. In the three or four district-level hospitals that used paper-based systems, ARV drugs were stored in the main district pharmacy for the respective hospital. Drug disbursement or transfers to the ART facility within these hospitals are documented on pharmacy stock cards; however, confirmation of whether all drugs collected from the main pharmacy were actually given to patients at the ART facilities was difficult to collect. ART facilities, likely hospitals, with this pharmacy set-up had substantial differences in ART retention estimates between pharmacy stock data and the clinic cohort report. Improved and simplified pharmacy records systems are needed in district hospitals to note drug transfers to other ART facilities more accurately and to ensure quality pharmacy data. The model of ARV drug dispensing, common in health centre pharmacies may be less prone to errors caused by drug transfers. Health centres usually store drugs in the nurse review room for direct drug dispensation to patients. The rooms have both ARV registers and pharmacy stock cards, simplifying data recording and, ultimately, leading to more accurate estimation of patients retained on ART.

Among facilities with electronic data systems, the pilot generally showed high levels of agreement of ART retention estimates between the cohort report generated electronically and the pharmacy stock records. This similarity is likely because of the accuracy of the data in the electronic system which in turn reduced variations between the two sources. The high level of agreement between the cohort report generated electronically and the pharmacy stock records may indirectly confirms previous findings of the accuracy of data in the electronic data system [7]. However, one ART facility, Mangochi Hospital, adopted an electronic data system in quarter three and showed quite low precision in that quarter. The results from Mangochi were likely to be due to the temporary challenges in transitioning from a paper-based data system to an electronic data system. Moreover, as Mangochi had the largest number of patients on ART, this finding may also indicate that the paper-based system may work effectively to a certain patient-number threshold beyond which accuracy may decrease, necessitating an electronic data system.

Addressing current challenges in management of pharmacy stock records could make this a viable approach to estimating patient retention in the future and would provide several advantages over current estimate techniques. First, estimation of ART retention based pharmacy stock records is more cost-effective in terms of healthcare personnel time and effort. Second, pharmacy stock records could help verify the accuracy, or identify weaknesses, in either paper- or electronic-based ART retention reports, providing an additional layer of monitoring and verification for ART programs. Lastly, improving pharmacy records for ART could have a trickle-down effect on improved general pharmacy record management by ensuring an uninterrupted supply of drugs which is a condition for life-long patient retention on ART and, contributing to health system strengthening overall.

The results of this pilot study should be viewed in light of the following limitations. First, pharmacy stock cards were missing in some facilities due to poor handover between pharmacy technicians, potentially leading to underestimation of ART retention. In other facilities, pharmacy records and ARV registers in facility clinic rooms were incomplete reducing their use in estimating patient retention. Moreover, in cases of low ARV stock levels, ARV clinics may divide and share individual bottles of ARVs, complicating calculations. But, as stock-out events were not reported during the study period, this probably did not affect the findings. Central hospitals had some patients on non-standard ARV regimens, but these patients could not be estimated from pharmacy stock cards as the estimation process was not feasible. As this was an observation pilot study, we did not conduct additional training on record management for the pharmacist or nurses prior to the study which would have improved the quality of pharmacy stock data and of retention estimates. Lastly, in case of large discrepancies in the estimates of patients retained on ART between the two sources of information, we were unable to determine the more accurate monitoring system, especially in paper-based facilities. However, we can hypothesise that in such situations the data quality might be affected in both monitoring systems, calling into question patient retention in estimates at the specific facility regardless of calculation method.

## Conclusions

Overall, the pilot study found that estimates of ART patients retained in care using pharmacy stock data were largely similar to patient retention estimates calculated using routine cohort report data collected from either paper- or electronic-patient registers. Among facilities using paper-based patient registers, using stock cards reduced the time needed to compile the data more than 5-fold, a dramatic decrease in the personnel time required for this routine reporting requirement. While electronic pharmacy management systems supported by well-trained personnel would be ideal, addressing current challenges in ARV stock management would likely make pharmacy stock cards a viable and cost-effective option for estimating patients retained on ART especially among facilities still using paper-based patient registries. Additional studies to explore the accuracy, feasibility and costs of using pharmacy stock data in other low-resource settings would strengthen these results and could lead to scale-up of this less time intensive method for patient retention estimation across sub-Saharan Africa.

## Competing interest

We declare that we have no conflict of interest.

## Authors’ contribution

ADH, RK, JMT conceived the study. HT, CF, ABS, AJ, SP contributed to the study design. HT drafted the manuscript. CF, ADH, RK, JMT, ABS, AJ and SP critically revised the manuscript. All authors gave approval of the final version to be published.

## Pre-publication history

The pre-publication history for this paper can be accessed here:

http://www.biomedcentral.com/1472-6963/12/210/prepub
